# Society Meetings

**Published:** 1896-01

**Authors:** 


### Society Meetings.

THE Medical Society of the County of Chautauqua will hold its semi-annual meeting at the Sherman House, Jamestown, N. Y., on Tuesday, January 14, 1896, at 11 o'clock, A. M., under the presidency of Dr. E. S. Rich, of Kennedy.

The secretary, Dr. C. A. Ellis, of Sherman, has sent out the following program : Report of interesting case, Dr. Morris N. Bemus, Jamestown, N. Y. Report and presentation of case of fracture of the skull, Dr. E. S. Rich, Kennedy, N. Y. The abuse of some narcotics, Dr. C. E. Lundgren, Jamestown, N.Y.; discussed by Dr. Edward Rood, Westfield, N. Y., and Dr. L. P. McCray, Clymer, N. Y. Criminal abortion, Dr. James Murphy, Sherman, N. Y. ; discussed by Dr. T. D. Strong, Westfield, N. Y., and Dr. E. A. Scofield, Bemus Point, N. Y. Intubation of the larynx, Dr. N. G. Richmond, Fredonia, N. Y. ; discussed by Dr. George E. Blackham, Dunkirk, N. Y., and Dr. E. C. Lyman, Jamestown, N. Y. ; Discussion on Pneumonia : (*a*) Pathology and Etiology, by Dr. J. C. Lewis, Panama, N. Y.; discussed by Dr. G. F. Smith, Sinclairville, N. Y., and Dr. J. W. Morris, Jamestown, N. Y. (*b*) Symptoms and diagnosis, by Dr. H. W. Davis, Falconer, N. Y.; discussed by Dr. V. M. Griswold, Fredonia, N. Y., and Dr. N. E. Beardsley, Dunkirk, N. Y. (*c*) Treatment, by Dr. Wm. M. Bemus, Jamestown, N. Y.; discussed by Dr. Laban Hazeltine, Jamestown, N. Y., and Dr. F. E. Lilley, Findleys Lake, N. Y.

A cordial invitation is extended to the medical profession of Chautauqua and adjoining counties.

### ITEMS.

At a recent meeting of the board of supervisors of the County of Chautauqua, a resolution was passed fixing a fee of three dollars with 25 cents mileage for examiners in lunacy in county cases.

The Jamestown Medical Society has already taken steps to defend its members against such an inadequate fee, and it is presumed that the County Medical Society will also take action on this subject at its forthcoming meeting.

The physicians of Chautauqua are combining in an association that has for its purpose the boycotting of "dead beats" and they will soon decline to render services for such characters without pay in advance. This timely action is commended to other medical organizations.

---

The Medical Society of the County of Erie will hold its seventy-fifth annual meeting at the rooms of the Buffalo Academy of Medicine, 619 Main street, on Tuesday, January 14, 1896, beginning at 9 o'clock A. M. On that occasion the society will celebrate its seventy-fifth anniversary, the arrangements of which are in the hands of the following committee: Dr. Lucien Howe, chairman; Dr. Charles G. Stockton, Dr. H. Mynter, Dr. J. W. Putnam and Dr. W. H. Gail.

Dr. Franklin C. Gram, the secretary, has prepared the following program: (1) Order of business, according to the by-laws. (2) Some unavoidable accidents of intubation, by Dr. Geo. F. Cott. (3) Short history of the society, by Dr. F. C. Gram. (4) Reading of special act legalising this society and voting upon resolution of incorporation. (5) Epochs in local medical history; short addresses by: (*a*) Dr. John Hauenstein, First uses of chloroform and ether in Buffalo. (*b*) Dr. J. B. Samo, Cholera epidemics in Buffalo. (*c*) Dr. C. C. Wyckoff, Establishment and early days of the Medical Department of the University of Buffalo. (*d*) Dr. John Cronyn, Establishment of Medical Department of Niagara University. (*e*) General discussion and closing business.

---

The Microscopical Club of the Buffalo Society of Natural Sciences, Dr. Frank J. Thornbury, president, announces the following program for 1896: January 13th—Geo. W. Rafter, M. Am. Soc. C. E., Rochester, N. Y., Lake Erie as a water supply for the towns on its borders; Herbert U. Williams, M. D., The parasite of malaria.

February 10th—Chauncey Pelton Smith, M. D., The protizoa, their etiological relation to cancer, demonstration ; Miss Edna Porter, Some common fungi. March 9th—Prof. F. G. Novy, Ann Arbor University, Michigan, subject to be announced. April 13th—Franklin W. Barrows, B. S., M. D., A microscopical study of nerve cells in hunger and fatigue, embryological exhibit ; Fred. C. Bush, B. S., Cutting and imbedding—most improved methods, notes on the use of formalin. May 11th—Annual meeting; report of the secretaries and treasurer ; president's address ; election of officers.

Meetings will be held in the Buffalo Library and Art Building, natural science lecture room, on the second Monday in each month and will be called to order at 8.15 P. M.

---

The St. Louis Academy of Medical and Surgical Sciences was organised November 6, 1895. The membership is limited to fifty. The following officers were elected for the ensuing year : President, Dr. Geo. W. Cale, Jr.; senior vice-president, Dr. James Moores Ball ; junior vice-president, Dr. Arthur E. Mink ; secretary, Dr. Emory Lanphear ; treasurer, Dr. Wellington Adams ; orator, Dr. Thomas O. Summers ; curator, Dr. George Howard Thompson.

---

The Medical Society of the State of New York will hold its ninetieth annual meeting at Albany, on Tuesday, Wednesday and Thursday, January 28, 29 and 30, 1896, under the presidency of Dr. Roswell Park, of Buffalo. The delegates and members should take cognisance of the fact that the meeting this year is to be held a week earlier than usual, the object of which is to secure better hotel accommodations than can be had during the first week in February.

---

The Western Association of Obstetricians and Gynecologists held its fifth annual meeting at Kansas City, Mo., December 27 and 28, 1895, under the presidency of Dr. J. E. Summers, Jr., Omaha, Neb.

---

The American Microscopical Society will hold its nineteenth annual meeting in the new Carnegie Library Building, Pittsburg, Pa., Tuesday, Wednesday, Thursday and Friday, August 18, 19, 20 and 21, 1896. A hearty welcome will be extended to all interested in the microscopical sciences. Applications for membership and titles of papers to be read at the meeting should be addressed to A. Clifford Mercer, M. D., president, Syracuse, N. Y., or to Wm. C. Krauss, M. D., secretary, 382 Virginia street, Buffalo, N. Y.

